# CRISPR-Cas Systems in the Cyanobacterium *Synechocystis* sp. PCC6803 Exhibit Distinct Processing Pathways Involving at Least Two Cas6 and a Cmr2 Protein

**DOI:** 10.1371/journal.pone.0056470

**Published:** 2013-02-18

**Authors:** Ingeborg Scholz, Sita J. Lange, Stephanie Hein, Wolfgang R. Hess, Rolf Backofen

**Affiliations:** 1 Genetics and Experimental Bioinformatics Group, Faculty of Biology, University of Freiburg, Freiburg, Germany; 2 Bioinformatics Group, Department of Computer Science, University of Freiburg, Freiburg, Germany; University of Florida, United States of America

## Abstract

The CRISPR-Cas (Clustered Regularly Interspaced Short Palindrome Repeats – CRISPR associated proteins) system provides adaptive immunity in archaea and bacteria. A hallmark of CRISPR-Cas is the involvement of short crRNAs that guide associated proteins in the destruction of invading DNA or RNA. We present three fundamentally distinct processing pathways in the cyanobacterium *Synechocystis* sp. PCC6803 for a subtype I-D (CRISPR1), and two type III systems (CRISPR2 and CRISPR3), which are located together on the plasmid pSYSA. Using high-throughput transcriptome analyses and assays of transcript accumulation we found all CRISPR loci to be highly expressed, but the individual crRNAs had profoundly varying abundances despite single transcription start sites for each array. In a computational analysis, CRISPR3 spacers with stable secondary structures displayed a greater ratio of degradation products. These structures might interfere with the loading of the crRNAs into RNP complexes, explaining the varying abundancies. The maturation of CRISPR1 and CRISPR2 transcripts depends on at least two different Cas6 proteins. Mutation of gene *sll7090*, encoding a Cmr2 protein led to the disappearance of all CRISPR3-derived crRNAs, providing *in vivo* evidence for a function of Cmr2 in the maturation, regulation of expression, Cmr complex formation or stabilization of CRISPR3 transcripts. Finally, we optimized CRISPR repeat structure prediction and the results indicate that the spacer context can influence individual repeat structures.

## Introduction

The RNA-based prokaryotic defense mechanism involves (i) an array of Clustered Regularly Interspaced Short Palindromic Repeats (CRISPR), made up of a leader, frequently palindromic repeated sequences with unique spacers located in-between, and (ii) a defining set of CRISPR-associated (Cas) proteins (see general reviews [1]–[7]. CRISPR-Cas systems are extremely diverse across different organisms, can be exchanged via horizontal gene transfer [8] and provide an adaptive immunity against invading phages and other genetic elements for the majority of archaea and many bacteria [9]–[11]. The CRISPR arrays are transcribed and subsequently processed into shorter RNA molecules (crRNAs) about 30–50 nucleotides (nt) long. The crRNAs interact with their respective Cas protein complexes to form a ribonucleoprotein (RNP), where they serve as guides to target mostly foreign DNA or RNA molecules for cleavage and degradation [1], [4], [12]–[15].

Currently, at least 45 families of Cas proteins have been identified [16], and the different types of CRISPR are associated with different subsets of these Cas proteins. These modules function independently and highly specifically with their respective crRNAs to affect CRISPR-Cas defense. Characterized examples include the CMR (*C*as *m*odule *R*AMP (repeat-*a*ssociated mysterious proteins)) and the CASCADE (*C*RISPR-*as*sociated *c*omplex for *a*ntiviral *de*fense) complexes of *Pyrococcus furiosus* and *E. coli*, respectively [17], [18]. By comparing phylogenies of common *cas* genes, repeat sequences and the architecture of CRISPR-*cas* loci, CRISPR-Cas systems can be categorized into types [15], [16], [19]. The most recent classification by Makarova *et al*. has defined three major categories of CRISPR–Cas systems, which can be further divided into at least ten subtypes and some chimeric variants [15], [19].

Endoribonucleases from various CRISPR-Cas systems (types I and III) are key players in crRNA maturation and are known to cleave repeats at distinct sequence and structure motifs leaving an 8 nt 5′ repeat handle (5′ tag): Cas6 [20]–[22]), Cse3 [17], [23], [24], recently renamed to Cas6e [15], and Csy4 [25], [26], recently renamed to Cas6f [15]. In contrast, type II systems use a tracrRNA and the host RNase III to process the arrays [27]. After cleavage, the crRNAs are trimmed by a poorly characterized ruler mechanism to fixed lengths [28].

CRISPR-Cas systems are not only highly diverse across species, but a single organism, such as the model cyanobacterium *Synechocystis* sp. PCC6803 (from here on *Synechocystis* 6803), can harbor complex clusters of distinctly different CRISPR loci. The photosynthetic cyanobacteria lack homologs to those Cas proteins commonly associated with the CASCADE complex in bacteria, but possess Cmr proteins instead. Many cyanobacteria and archaea share the almost exclusive presence of proteins from the Csc family (for CRISPR/Cas subtype cyano), characteristic for subtype I-D CRISPR-Cas systems [19]. Despite these unique properties, cyanobacterial CRISPR-Cas systems are only poorly characterized. *Synechocystis* 6803 harbors three CRISPR arrays on its 103,307 nt plasmid pSYSA, each annotated with distinctly different sets of associated *cas* genes. CRISPR1 is classified as subtype I-D, whereas CRISPR2 and CRISPR3 are type III systems [15], [19]. Representatives of type III systems have been well characterized in archaea [18], [21], [22], [29]–[33], whereas only a single such system, that of *Staphylococcus epidermidis*, has been studied experimentally in a bacterial host [28].

Vital to a successful CRISPR-based defense is the expression and accurate processing of mature crRNAs; by analyzing different aspects of the expression and maturation of crRNA, we determined that these three CRISPR loci in *Synechocystis* 6803 are highly distinct and independent in their processing mechanisms. We combined (i) assays of transcript accumulation, (ii) functional knock-out experiments of selected Cas and one Cmr protein, (iii) high-throughput transcriptomics, and (iv) in-depth computational analyses of RNA structure to elucidate significant processing features. Throughout, our results highlight the notable differences and independent processing pathways of these CRISPR-Cas systems.

## Results

### Characteristics of the *Synechocystis* 6803 CRISPR-Cas Systems on pSYSA

**Figure 1:**
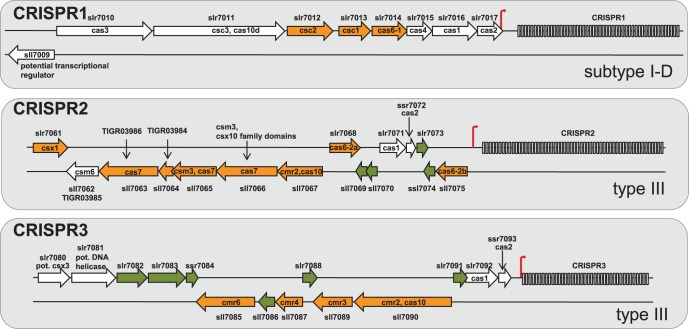
Organisation of the three CRISPR-*cas* systems on plasmid pSYSA of *Synechocystis* 6803. Several *cas*-genes are located upstream of each of the three CRISPR arrays. Arrows in green represent genes coding for hypothetical proteins and arrows in orange illustrate *cas*-genes from the RAMP family. Experimentally mapped start sites of transcription (TSS) are marked by thin red arrows. Direct repeats are symbolized by narrow rectangles. For selected genes, synonymous designations are given but in the following we use the nomenclature introduced by Makarova *et al*. [15], [19].

The plasmid pSYSA of *Synechocystis* 6803 is a large, extrachromosomal element that is almost entirely devoted to three different CRISPR-Cas systems, CRISPR1-3, located on the forward strand. Each repeat-spacer array is adjacent to a distinct set of associated *cas* genes (**Figure 1** and **Table 1**). Among CRISPR1 genes are homologs to *cas3* (*slr7010*) and *csc3/cas10d* (*slr7011)*, which serve as markers of CRISPR subtype I-D [15]. In contrast, CRISPR2 and CRISPR3 resemble type III CRISPRs, indicated by the presence of *cmr2/cas10* homologs. Other subtype-specific markers such as *csm2* or *cmr5*, however, are missing.

**Table 1:** Characteristics of the three CRISPR (1, 2, 3) arrays present in *Synechocystis* 6803.

CRISPR	Subtype [15], [19]	repeat sequence	TSS	Leaderlength (nt)	No. of spacers	Spacer length (nt)	Identical spacer-repeat units
1	I-D	CTTTCCTTCTACTAATCCCGGCGATCGGGACTGAAAC	16097	213	49	31–41	10–11, 30/31–32/33, 40–41
2	III	GTTCAACACCCTCTTTTCCCCGTCAGGGGACTGAAAC (GTTCAACACCCTCTTTTCCCCGT**T**AGGGGACTGAAAC )	68374	125	56	34–46	6/7–8/9, 37/38–39/40
3	III	GTCTCCACTCGTAGGAGAAATTAATTGATTGGAAAC (GTCTCCACTCG**C**AGGAGAAATTAATTGATTGGAAAC )	90104	1	38	35–47	none

For each CRISPR, the repeat sequence and single occurring mutant variants near the 3′ end are given in brackets (the widely conserved [8] 3′ end GAAAC is underlined, and the mutated nucleotide is in boldface). The position of the TSS in the plasmid pSYSA defines the 5′ leader as the region between it and the first repeat. The number of spacers and the range of their lengths are given in addition to spacer-repeat unit duplications. Consecutive identical spacer-repeat units are separated by a dash, whereas duplicated unit pairs are united by a slash.

Three potential Cas6 endoribonuclease genes are located on pSYSA: *slr7014 (cas6-1*), adjacent to CRISPR1, *slr7068 (cas6-2a)* and *sll7075 (cas6-2b*), both adjacent to CRISPR2 (**Figure 1**). No Cas6 homolog is associated with CRISPR3. Their pairwise protein sequence similarity and their similarity to the functionally characterized Cas6 homolog of *Pyrococcus furiosus* is very low, ranging between 6–17% identical amino acid residues.

According to the previously published sequence [34], CRISPR1-3 consist of 49, 56 and 38 repeat-spacer units per locus (each with an additional final repeat). However, during a recent resequencing analysis of the laboratory substrain *Synechocystis* sp. “PCC-M” used here, a 33 repeat-spacer units deletion in CRISPR1 and a shorter deletion in CRISPR2 were observed [35]. Consequently, only 16 crRNAs were expressed from the CRISPR1 locus and 54 from the CRISPR2 locus. The spacer sequences differ in length from 31–47 nt and with the exception of a few identical spacers within CRISPR1 and CRISPR2 they are all unique. Identical single repeat-spacer units and pairs of two adjacent repeat-spacer units appear in a consecutive manner in CRISPR1 and CRISPR2 (**Table 1**).

### The CRISPR Arrays are Highly Expressed with Single Transcriptional Start Sites

**Figure 2:**
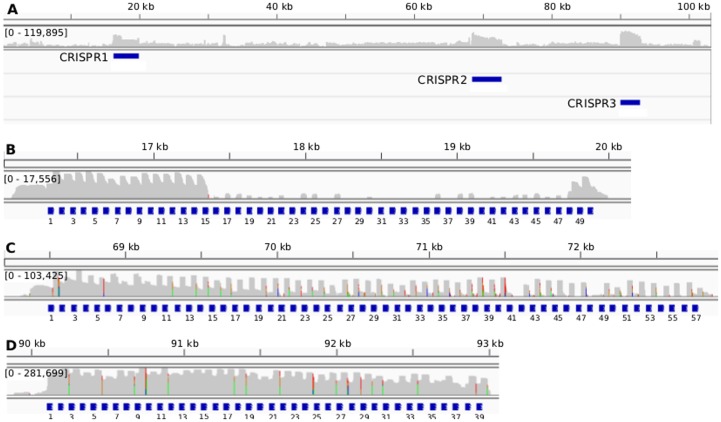
High expression levels of CRISPR-derived RNA on the pSYSA plasmid. (A) depicts the read coverage in log-scale (grey track) across the entire plasmid and the locations of the CRISPRs 1 to 3 are annotated as blue bars. All three CRISPRs are the most abundantly expressed loci on the plasmid. (B–D) show the expression profiles for CRISPRs 1 to 3, in that order. The reads have been filtered to reduce noise and the grey tracks in (B–D) depict their coverage profiles in log-scale. The numbers in the square brackets represent the absolute read number range; CRISPR3 is clearly the most abundantly expressed in comparison to the other two. The repeats are marked below by blue squares with their occurrence number. Due to the consecutive duplications of repeat-spacer units in CRISPR1 and CRISPR2 (**Table 1**), a unique mapping was impossible for these spacers so that their coverage appears identical. Moreover, CRISPR2 and CRISPR3 show a terminal processing despite the fact that there is no downstream repeat.

A transcriptome analysis revealed an extremely high level of CRISPR-derived RNA transcripts, especially in comparison to other loci on the pSYSA plasmid (**Figure 2A**). CRISPR3 RNA was most abundant with more than two million reads, almost 7 and 19 times more than CRISPR2 and CRISPR1, respectively. Only very few reads (a total of 110, 60, and 1430) mapped to the reverse strand; the majority mapping to the forward strand of CRISPR arrays 1 to 3. This suggests only a very minor effect of technical bias introduced by the reverse transcription and sequence analysis.

In **Figure 2B–D**, we present close-ups of the read coverage for each of the CRISPR arrays. The reads in each of the close-up views have been filtered to reduce noise mainly due to multiple mappings of repeat sequences. The CRISPR loci had a greater read coverage at the 5′ end in comparison to the 3′ end, which was also observed in e.g. Hale *et al*., 2012 [29]. Despite the generally high abundance of reads for all three CRISPRs, we noticed a lack of coverage corresponding to the repeat-spacer units 15–47 in CRISPR1 (**Figure 2B**), which results from a 2,399 bp deletion in this region, encompassing the repeat-spacer region 15–47 [35].

To define exact transcript boundaries of the CRISPR arrays, we mapped the TSS from the data in Mitschke *et al*., 2011 [36]. The precursor RNAs for CRISPR1-3 originated from one TSS each. The TSS is located at position 16097 for CRISPR1, 68374 for CRISPR2, and 90104 for CRISPR3, resulting in transcribed 5′ leaders of lengths 213, 124, and 1 nt, respectively (**Table 1**).

### Characterization of CRISPR-derived crRNAs and Processing Intermediates

**Figure 3:**
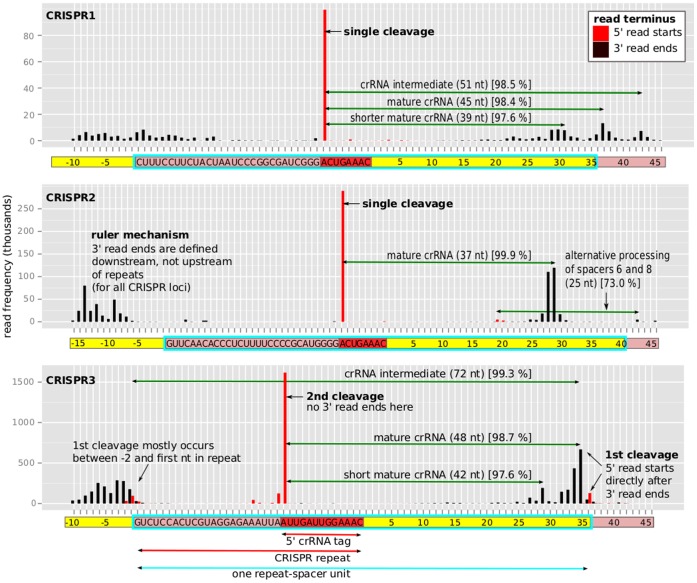
Frequency of read termini shows clear cleavage sites and distinct processing features. The number of reads (y-axis) starting (red) or ending (black) at a position relative to the closest repeat (x-axis) across an entire CRISPR locus illustrates the CRISPR maturation products (for RNA-seq dataset A). The repeat sequence is indicated in the pink+red, the 5′ crRNA tag in the red, and the relative position in the spacer in the yellow rectangles, respectively (x-axis). One repeat-spacer unit is framed by the thick cyan square (due to different spacer lengths, the mode is illustrated). The green arrows correspond to the most abundant reads, i.e. the processed mature crRNAs or intermediate products. Albeit spacers of different lengths, we clearly see the ruler mechanism as the mature crRNA is trimmed to a fixed. We identified the location of the accumulating reads by giving the percentage of reads in the respective read-length category that map to the illustrated location (square brackets). For CRISPR3, the first cleavage site is in the spacer (not in the repeat), supported by two observations (i) reads only end at the cleavage site in the spacer, not in the repeat, (ii) there is no accumulating RNA species that spans across the cleavage site in the spacer, whereas the 72 nt intermediate spans across the 13 nt cleavage site. CRISPR1 and CRISPR2 display only single cleavage sites and crRNAs are subsequently trimmed to their final length. CRISPR1 and CRISPR3 both have a second, less abundant mature crRNA transcript, which is exactly 6 nt shorter, whereas CRISPR2 only has one accumulating product. Note: Reads that appear 1–3 nt shorter are due to unknown read ends because of the poly(A) tails in the RNA-seq protocol.

In agreement with their characterization as distinct types of CRISPR-Cas systems, processing intermediates and mature crRNAs of different characteristic lengths were observed (**Figures 3** **and** **4**). We established cleavage sites and the boundaries of accumulating transcripts by counting the total number of 5′ and 3′ read starting and ending positions, relative to the closest direct repeat (summarized across all repeats across one array), using the RNA-seq dataset A (**Figures 3** **and** **4**). Note that due to the ligated poly(A) tails in the RNA-seq protocol, 3′ read ends were not well-defined for sequences ending in A’s, leading to staggered peaks. The repeat cleavage sites were most obvious with clear peaks of 5′ read starts giving rise to the well-published 5′ crRNA tags. The 5′ tags of CRISPR1 and CRISPR2 were identical (ACUGAAAC) and their length of 8 nt is in agreement with previous results [17], [18], [20]–[22], [26], [28], [37]–[39]. The 5′ tag of CRISPR3 is unusual by having a length of 13 nt. Its sequence AUUGAUUGGAAAC, however, exhibits similarities to many other published CRISPR repeats. For example, 9 out of the 12 published repeat classes [40] had a conserved AUUG prefix for the last 8 nt of their respective sequences, a motif that is duplicated in this CRISPR3 13 nt tag. Concerning the number of observed cleavage events, CRISPR1 and CRISPR2 displayed only single cleavage sites within their repeats, whereas CRISPR3 was processed with a double cleavage activity. Interestingly, the first cleavage occurred at the 5′ end of the repeats, mostly within the spacers. This result is supported by two observations (**Figure 3**): (i) 3′ read ends in the spacers were immediately followed by 5′ read starts, defining a clear cleavage site, which was not the case for the cleavage site at the 13 nt tag and (ii) there is no accumulating RNA species that spans across the cleavage site in the spacer, whereas the 72 nt intermediate spans across the 13 nt cleavage site.

**Figure 4:**
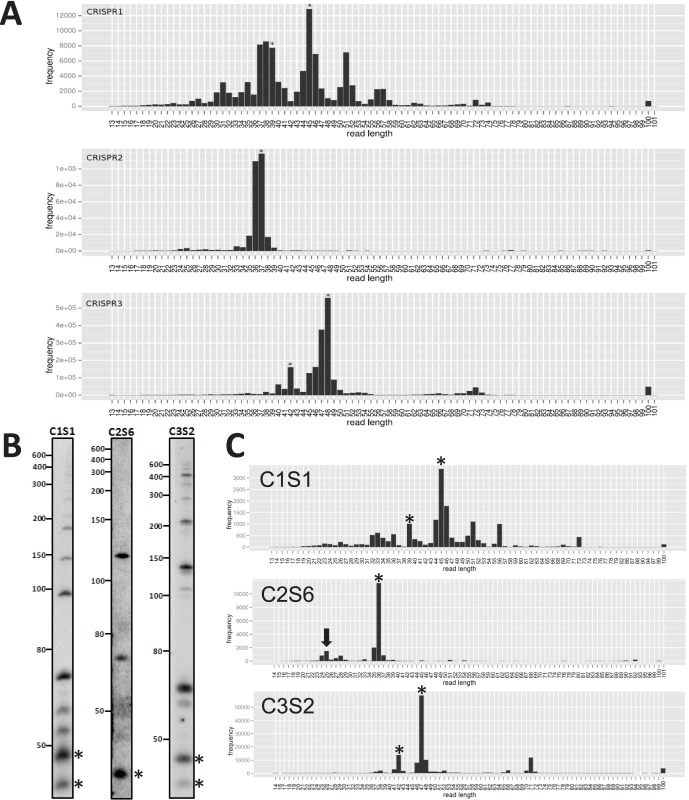
Accumulation of CRISPR RNA indicate lengths of mature crRNAs and intermediates. (A) Read frequencies (y-axis) for each CRISPR loci, computed from RNA-seq dataset A. Read lengths are given on the x-axis, whereby it is important to note that the poly(A) tails of the RNA-seq protocol obscure read ends such that lengths of reads ending in A’s cannot be determined exactly. (B) Northern hybridization using a synthetic oligonucleotide probe against spacer 1 of CRISPR1 (C1S1), spacer 6 of CRISPR2 (C2S6) and spacer 2 of CRISPR3 (C3S2) to identify bands of accumulating transcript species. (C) Specific read frequencies for spacer 1 of CRISPR1 (C1S1), spacer 6 of CRISPR2 (C2S6) and spacer 2 of CRISPR3 (C3S2), for which the hybridization pattern is depicted in panel (B). The atypical accumulation of a transcript of 25 nt for spacer 6 of CRISPR2 is highlighted by the arrow. Mature crRNAs are indicated by asterisks (all panels).

To further characterize the accumulating transcripts for each CRISPR locus, we calculated (from the filtered sets **Figure 2B–D**) the percentage of reads that mapped to the locations indicated in **Figure 3** out of all reads with the respective characteristic lengths (percentage in square brackets and 1–2 nt position-specific variation was allowed). The high percentages gave convincing evidence that the indicated locations are correct. The most probable mature crRNAs are 45 and 39 nt for CRISPR1, 37 nt for CRISPR2, and 48 and 42 nt for CRISPR3. Notice that for CRISPR1 and CRISPR3, two accumulating species of mature crRNAs existed, which were both 6 nt different in size and the longer transcript was more abundant (both observations were previously seen in *Pyrococcus furiosus* and *Staphylococcus epidermidis* RP62a [28], [29]). Despite the common difference in mature crRNA lengths for CRISPR1 and CRISPR3, however, other distinct features existed: In all Northern hybridizations, double bands were observed for CRISPR3 (**Figures 4B** **,** **5B** **,** and **6**), which indicated two distinct lengths (∼6 nt apart) for each accumulating transcript species; whereas for CRISPR1 this was not observed. For CRISPR1, accumulating transcripts of multiple lengths existed, all shorter than one repeat-spacer unit (∼71 nt), alluding to a step-wise trimming between two repeat-based cleavage sites.

**Figure 5:**
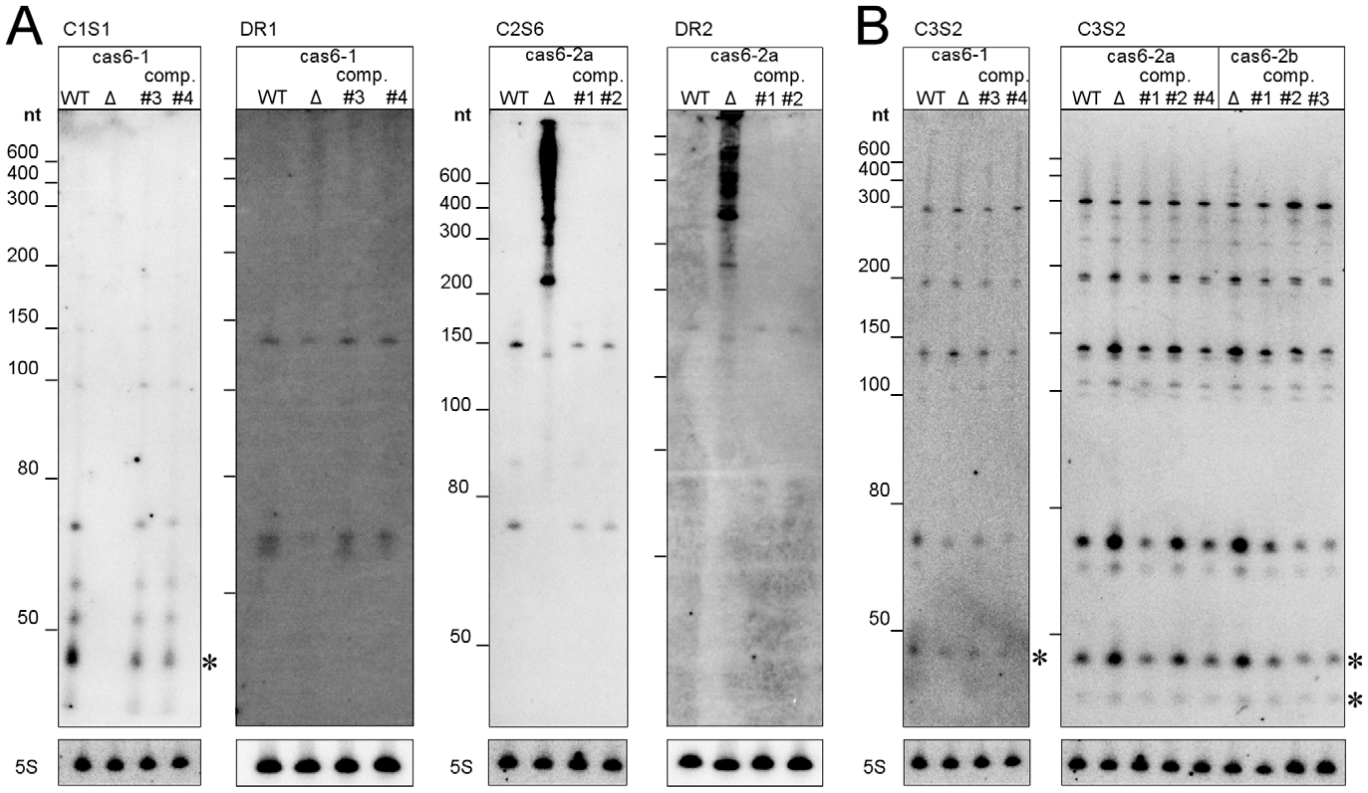
Impact of mutants on the accumulation of CRISPR-derived crRNAs in *Synechocystis* 6803. (A) Knock-out mutants of genes *cas6-1* and *cas6-2a* were tested for the accumulation of CRISPR1- or CRISPR2-derived transcripts by hybridization using a synthetic oligonucleotide against spacer 1 of CRISPR1 (C1S1) or spacer 6 of CRISPR2 (C2S6), or against the direct repeats. Reintroduction of a C-terminally FLAG-tagged *cas6-1* or *cas6-2a* gene on a replicating plasmid vector restored the wildtype pattern of CRISPR RNA accumulation (lanes labelled “comp,” different clones were tested). Lanes with RNA from the respective knock-out mutant are labeled by a Δ symbol. (B) Neither the *cas6-1* nor the *cas6-2a* or *cas6-2b* knock-out affected the accumulation of CRISPR3-derived transcripts, indicated by hybridization of a probe against spacer 2 of CRISPR3 (C3S2). Hybridization of 5S rRNA is shown for control. Mature crRNAs are indicated by asterisks.

**Figure 6:**
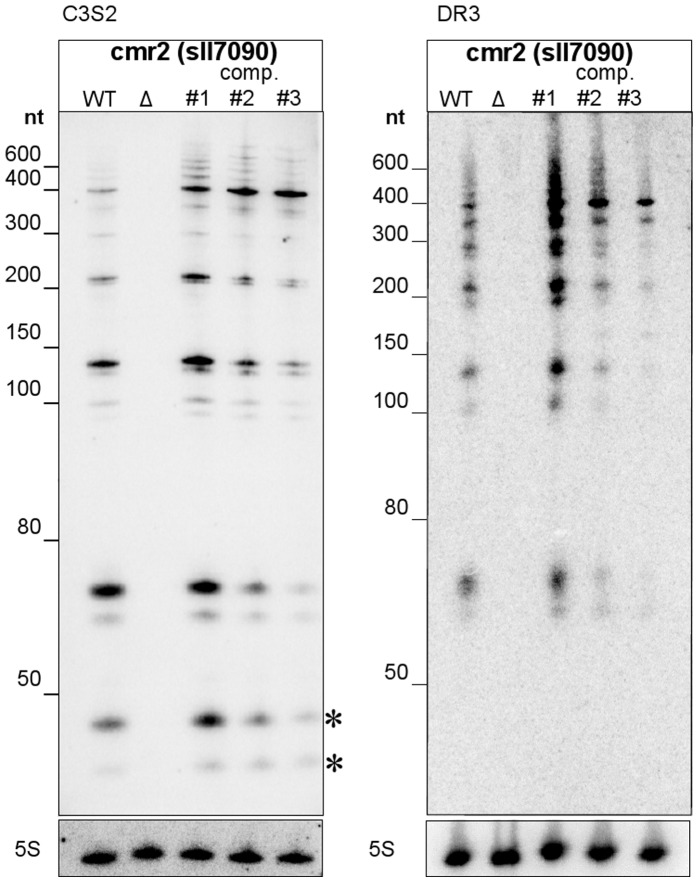
The Cmr2 protein encoded by gene *sll7090* is a major factor for the expression of CRISPR3. The effect of its knock-out mutation (Δ) on the accumulation of CRISPR3-derived crRNAs, compared to wildtype (WT), is shown, together with the complementation by expressing a FLAG-tagged version of Cmr2 from a replicating plasmid *in trans* (“comp.”, three clones, #1–#3). A synthetic oligonucleotide against spacer 2 of CRISPR3 (C3S2) or against the direct repeat was used for hybridization. Mature crRNAs are indicated by asterisks.

Despite the varying lengths of the spacers, all crRNAs accumulated to these fixed characteristic lengths, which further supports the ruler mechanism published for the Csm and Cmr systems [28], [29]. Moreover, the final repeat for all loci was cleaved at the usual position and there existed a notable accumulation of a 3′ terminal transcript downstream from the last repeat for only CRISPR2 and CRISPR3 (**Figure 2C–D**). These transcripts were of equal length with their respective mature crRNAs, albeit no second 3′ repeat sequence; not even a partial, or a mutated repeat sequence could be detected. These terminal potential crRNAs indicate that the 5′ repeat is the anchor of the ruler mechanism and that this measured crRNA accumulation is independent of a subsequent cleavage in the downstream repeat. This was not observed for CRISPR1, which further supports the previously mentioned step-wise 3′ trimming.

In summary, while the described processing patterns shared previously published common features, detailed evidence suggests distinctly different pathways.

### Mutational Analyses Suggest the Involvement of Distinct Cas6 Endoribonucleases in the Maturation of CRISPR1 and CRISPR2

An important protein involved in processing CRISPR precursor transcripts is the Cas6 endoribonuclease, as demonstrated in *Pyrococcus furiosus* [20]–[22], [30], *Sulfolobus solfataricus* [39], and *Staphylococcus epidermidis* RP62a [28]. Therefore, we generated knock-out mutations of putative *cas6* homologs in *Synechocystis* 6803 to experimentally establish their involvement in CRISPR maturation. When judged by a spacer-specific probe, the mutation of *slr7014* (*cas6-1*) led to a loss of crRNA accumulation (**Figure 5A**). In contrast, the more sensitive hybridization against a repeat-specific probe revealed the presence of CRISPR1-derived transcripts, however, precursors with a higher molecular weight were relatively more abundant than shorter products (**Figure 5A**). These effects were completely abolished in the complementation experiment, verifying the knock-out of *cas6-1* as causative. Two other potential endoribonuclease genes, *cas6-2a* and *cas6-2b,* are both located in close proximity to CRISPR2. The *cas6-2a* knock-out mutation yielded a very distinct processing pattern: CRISPR2 precursor transcripts were not processed to less than 200 nt. Instead, all longer precursors accumulated to very abundant amounts, both when hybridized by a spacer-specific and by a repeat-specific probe (**Figure 5A**). Again, the complementation experiment verified that these effects were caused specifically by the knock-out of *cas6-2a* (**Figure 5A**). Despite these strong effects on transcript accumulation for CRISPR1 or CRISPR2, both mutations did not affect CRISPR3-derived transcripts (**Figure 5B**). These *in vivo* observations thus support specific endoribonuclease activities for the Cas6-1 and Cas6-2a proteins in the maturation of CRISPR1- and CRISPR2- derived crRNAs, respectively, which is also in agreement with their respective location (**Figure 1**).

### Accumulation of CRISPR3-derived crRNAs is Affected in a Cmr2 Knock-out Mutation

In contrast to CRISPR1 and CRISPR2, the accumulation and processing of CRISPR3-derived crRNAs was not affected by the knock-out mutation of either *cas6-1* or *cas6-2a,* and also not by the *cas6-2b* knock-out **(** **Figure 5B** **)**. The lack of an obvious candidate for a processing endonuclease for CRISPR3 appeared puzzling, but is consistent with the absence of a known endoribonuclease gene close to CRISPR3. Another noteworthy protein, however, is Cmr2, which has been predicted to have a nuclease domain of the histidine, aspartic acid (HD) family [41]. In fact there is a gene, *sll7090*, among the *cas* genes associated with CRISPR3 that is a possible *cmr2* homolog. Therefore, we generated a knock-out mutant of this *cmr2* gene to elucidate its function in this system. Indeed, the knock-out of *cmr2* strongly affected the accumulation of transcripts from CRISPR3 (**Figure 6**). A complete loss of precursor and processed RNA molecules was observed; this loss was judged by hybridization against a probe specific for spacer 2 and a probe against the direct repeat, whereas, the loss was reversed by the overexpression of *cmr2* from a plasmid vector (**Figure 6**).

### Stability of CRISPR3-derived crRNAs may be Dependent on Spacer Structure

**Figure 7:**
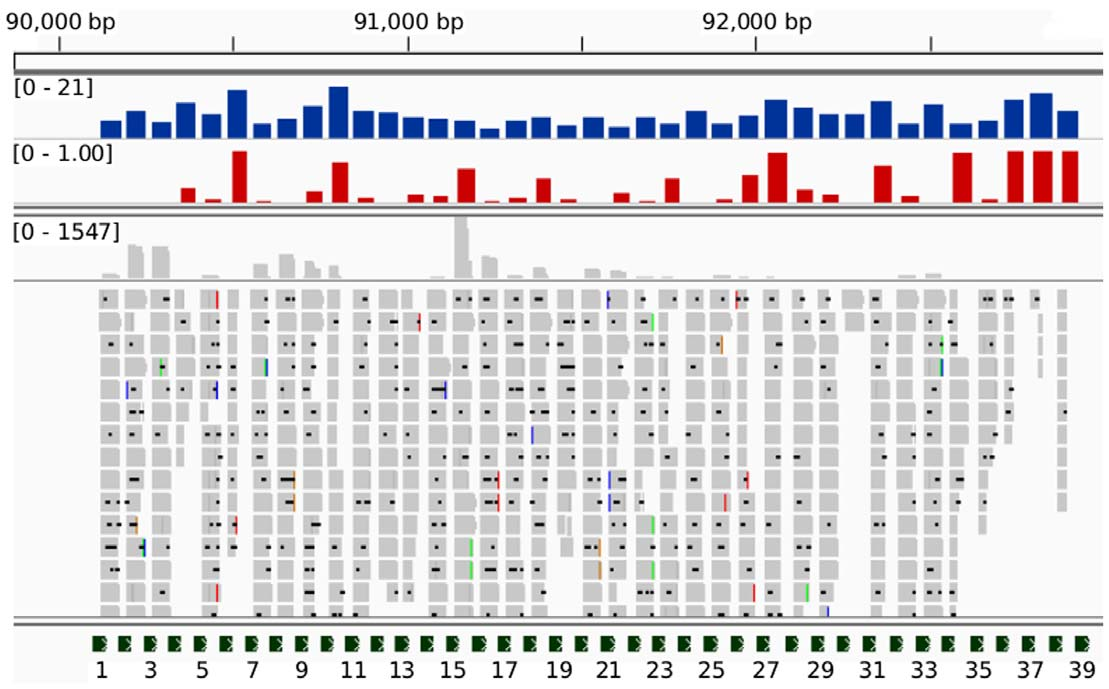
A significant relationship between the ‘structuredness’ and accumulation level of individual crRNAs. The degradation of mature crRNAs correlates with spacer ensemble energies with a Pearson’s correlation coefficient r = 0.56 and p = 0.00025 (RNA-seq dataset B). Depicted is the CRISPR3 locus on the chromosome of *Synechocystis* sp. PCC 6803 with the following tracks: (blue) The absolute ensemble energy of the spacer sequence as determined by RNAfold (greater values correspond to more stable structures); (red) the normalized degradation profile of previously processed crRNA; (grey) sequence reads corresponding to degraded or full-length mature crRNA; (green) the CRISPR repeat sequence locations. Some crRNA positions remain full-length (grey track), whereas other positions are degraded. We have selected only reads that correspond to mature crRNAs. Reads that cover two spacers were excluded for this analysis since they correspond to crRNA precursors.

We observed vast differences in the processed crRNA abundancies across the CRISPR arrays (note that the log-scale reduces the visible differences in **Figure 2**). Given that each CRISPR array has only one TSS and is thus transcribed as one transcript, no obvious reason for major differences in accumulation exists. This variability could be partially explained by the stability of the crRNA-Cas protein complexes: Highly structured crRNA could obstruct their formation leading to crRNA degradation. To test this idea, we compared the ratio of degraded products with respect to full-length crRNA to different structural properties of the CRISPR array. The most convincing correlation between degradation and RNA structure was seen in the ensemble energy of the separate spacer sequences (**Figure 7**, blue track) with a Pearson’s correlation coefficient of 0.56 (p = 0.00025). High ensemble energies correspond to spacers that can form more stable secondary structures. This indicated a strong relationship between the “structuredness” of the spacer and the degradation ratio of previously processed crRNA: more stable structures could lead to a higher rate of degradation (note that we give the absolute ensemble energy values and that in reality a negative correlation exists, due to negative energies). More precisely, all spacers with an ensemble energy below −15 kcal/mol had the highest degradation ratios. This result was achieved for the smaller RNA-seq dataset B. Albeit the statistically significant correlation for the larger dataset A at r = 0.38 and p = 0.018, the correlation in this set is not as convincing, which is likely due to the differences in the datasets: In dataset A, only about 4% of the reads where short enough to be considered as degradation products. It is unlikely that the signal was strong enough to be detected in this minor subset of reads, whereas in dataset B, the ratio of possible degradation products in comparison to non-degraded reads was much higher (see grey track in **Figure 7**). CRISPR1 and CRISPR2 could not be analyzed for correlation to structuredness because not enough reads mapped to these loci in dataset B.

### The Surrounding Spacers Influenced Repeat Structure Prediction

**Figure 8:**
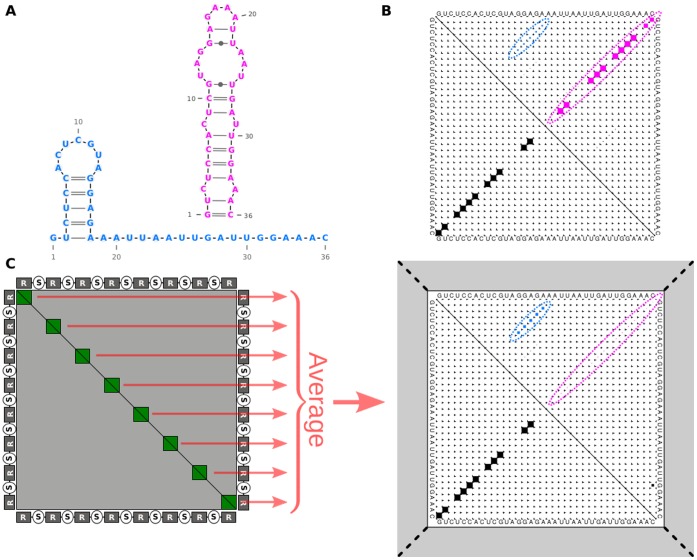
Structural analysis of CRISPR3 repeats: Comparison of structures resulting from the commonly used MFE prediction to our CRISPR-specific context-based approach. (A) The two most stable structure candidates; the MFE structure is in magenta. (B) The base-pair probability matrix, as computed by RNAfold [55], for the repeat sequence where the MFE structure is in the lower triangle and the two structures from (A) are clearly marked in the upper triangle. (C) Our approach: repeat structure in context. To analyze the influence of the context, we calculated the base-pair probability matrix for the complete array (R = repeat, S = spacer). The preferred structure in the context was determined by averaging the sub-matrices associated with the repeats. When the repeat was folded in its sequence context, the magenta structure nearly disappeared and the blue structure, which looks more like other known CRISPR structures, was more probable.

**Figure 9:**
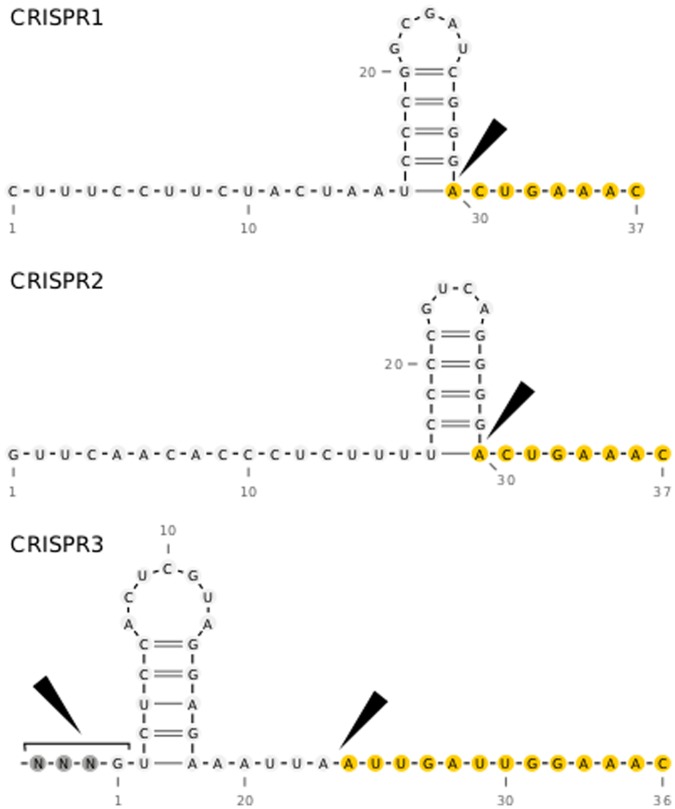
Predicted CRISPR repeat structures using our CRISPR-specific prediction approach that includes influencing context sequences. The black wedges indicate cleavage sites derived from the RNA-seq data and the yellow circles mark the 5′ repeat sequence tag of the mature crRNAs. The 5′ tags for CRISPR1 and CRISPR2 had the frequently published length of 8 nt. CRISPR3 was cleaved twice, first at the end of the spacer and second in the middle of the repeat leaving a novel-length 13 nt tag.

The general practice in the search for the functional CRISPR repeat structure is to compute the minimum free energy (MFE) structure of a single repeat sequence. The repeat is not transcribed as a single unit, however, but is located on a transcript in the context of other spacers and repeats. These flanking sequences can have a vast impact on the actual structure so that sub-optimal repeat structures could be preferred over the MFE structure. Although the MFE prediction is frequently correct due to highly stable stem-loop structures with many GC base-pairs, for example in *E. coli* [17], we show that this procedure may not always be accurate. Our structure prediction approach, tailored specifically to CRISPR features, includes the entire array sequence and determines the most stable structure formation within that context (illustrated in **Figure 8**). With this approach, we identified a repeat structure for CRISPR3 (blue structure in **Figure 8A**) that resembles native CRISPR structures [24], [25], [38], [42] much more closely than the MFE structure (magenta structure in **Figure 8A**). Whereas, for CRISPR1 and CRISPR2, the repeat MFE structure was also the most probable within its context. All final predicted structures are given in **Figure 9**.

## Discussion

### A Complex Cluster of Three CRISPR-Cas Systems on a Single Plasmid

Our combined experimental and computational results describe three CRISPR-Cas systems on plasmid pSYSA, each with an independent and unique set of associated proteins and a distinct processing pathway. Recently, it was shown that diverse defense systems are frequently clustered in prokaryotic genomes [43], which is pronouncedly true for the pSYSA plasmid of *Synechocystis* 6803 as several toxin-antitoxin systems are encoded on it, together with the here described CRISPR1-3. This apparent focus and variability in survival mechanisms in combination with a lack of knowledge on cyanobacterial CRISPR systems makes this an interesting plasmid to study in depth.

The maturation of crRNAs and precursor processing is essential to the function of the CRISPR-cas system [17]. Accordingly, we investigated the following key aspects: (i) annotation and characterization, (ii) expression, (iii) array processing patterns, (iv) identification of Cas proteins involved in crRNA maturation, (v) crRNA stability, and (vi) repeat structure motifs.

The three CRISPR-Cas systems were named CRISPR1-3 and are associated with distinct sets of associated Cas proteins, classified as a subtype I-D for CRISPR I and type III for CRISPR2 and CRISPR2; the latter two could not be classified into specific subtypes [15], [43].

High-throughput transcriptomics and molecular assays illustrated that transcripts from all CRISPR arrays were highly abundant, especially in comparison to other loci on the pSYSA plasmid. Mapping of transcription start sites gave rise to transcribed leaders for CRISPR1 and CRISPR2, but the TSS for CRISPR3 was only one nucleotide upstream of the first repeat. It is unknown whether this lack of a leader could affect new spacer acquisition; however, the array was evidently processed.

A more detailed analysis determined the length and locations of accumulating transcripts, identifying possible mature crRNA sequences, which were disproportionately abundant and thus clearly visible: 39 and 45 nt for CRISPR1, 37 nt for CRISPR2, and 42 and 48 nt for CRISPR3. In agreement to our results, the accumulation of two distinct crRNA species with 6 nt difference and their incorporation into the protein complex was shown previously, where the longer species was also the more dominant [28], [29]; in contrast, only a single mature crRNA accumulated for CRISPR2.

In addition, the most frequent 5′ and 3′ read end mapping locations gave a detailed insight into cleavage sites and processing patterns and especially highlighted the fact that the crRNAs from each locus must have been generated by distinct pathways. CRISPR1 had many accumulating transcript species all shorter than one repeat-spacer unit indicating a possible step-wise trimming mechanism from the 3′ end (arising from the cleavage site in the downstream repeat), whereas CRISPR2 and CRISPR3 crRNA maturation seemed to be independent of a downstream repeat. CRISPR3 showed a double-cleavage mechanism where the first cleavage occurred in the spacer (or at the 5′ end of the repeat); the second cleavage in the repeat generated a crRNA 5′ tag of an unusual 13 nt. Whereas, CRISPR1 and CRISPR2 displayed single repeat cleavages generating the usual 8 nt tag [17], [18], [20]–[22], [26], [28], [37]–[39]. Moreover, for all CRISPRs, we observed the measured trimming of the crRNAs to fixed characteristic lengths, despite the variability in spacer lengths. This further supports the recently published and poorly understood ruler mechanism as a post-processing step after the initial repeat-guided cleavage [28].

In spite of transcripts arising from single TSS, mature crRNAs accumulated to significantly different abundancies implying differences in their stabilities. Our computational analysis of CRISPR3 transcript accumulation indicated that spacers forming more stable structures are linked to higher degradation rates of the crRNA sequence. A similar observation has recently been reported for the crRNAs derived from CRISPR locus C in *Sulfolobus solfataricus*, where those crRNAs with the potential to fold into the more stable structures were clearly less abundant than those with only modest folding propensity [44]. Interestingly, the studied *Sulfolobus solfataricus* system is of CRISPR subtype III-B, similar to the CRISPR3 of *Synechocystis* 6803 studied here. Thus, the different quantities of mature crRNAs could be due to their different loading efficiencies into the CMR complex. A highly structured crRNA could prevent or delay the RNP complex formation and thus lead to a lack of protection and consequently higher rates of degradation. Therefore, the more efficient spacer is one that remains mostly unstructured.

Some Cas endoribonuclease proteins are known to bind to a hairpin motif in the repeat [24], [37]; we determined that the most probable repeat structure can depend on the surrounding spacer sequences. To predict the most probable repeat structure, we developed a CRISPR-specific repeat prediction method that calculates probabilities regarding the entire array and it delivered results superior to the commonly used MFE-based prediction; our identified repeat structure for CRISPR3 resembles native CRISPR structures much more closely than the MFE structure [24], [37], [40]. This suggests that the context could influence the individual repeat structure and thus also processing efficiency, however, we could not fully resolve this question with the available data.

Recently, it was demonstrated that a hairpin structure is important for Cas6-dependent processing in the type III CRISPR/Cas system of *Staphylococcus epidermidis* [28], which is in agreement with our predicted hairpin structures for CRISPR1 and CRISPR2. In further agreement to recent literature, the observed cleavage site is at the right-hand base of the hairpin structures, both cutting between CG and UA base-pairs. For each structured repeat published, cleavage has always occurred just below the last CG base-pair in the stem of the hairpin [17], [24], [28], [37], [38]. Furthermore, our predicted structures contain a majority of GC base-pairs (11 out of 15) and the G is on the right side of the stem (3′ end) in all except one instance, which is also true for those previously published structures. This G side of a stem seems to be important for recognition and cleavage [28]. Despite the obvious similarities between the CRISPR1 and CRISPR2 hairpin structures and their identical 5′ tag, these systems display a highly specific binding of their respective Cas6 proteins and distinct processing patterns of accumulating transcripts. Therefore, structure and motif similarity does not automatically infer identical processing mechanisms.

### Proteins Involved in CRISPR Precursor Maturation in *Synechocystis* 6803

In past work, the Cas6 endoribonuclease has been identified as the main player in the CRISPR RNA processing pathways in different organisms [17], [20], [25]. The extent to which the effects of Cas6-like proteins can be generalized, however, has not been fully resolved. In this work, we found that the accumulation of crRNAs for CRISPR1 and CRISPR2 of *Synechocystis* 6803 depended on distinct Cas6 homologs.

Despite the fact that CRISPR3-derived RNA accumulated to high quantities and was evidently processed, its maturation was Cas6 independent: None of the three identified Cas6 homologs had an effect on CRISPR3 transcript accumulation. Given that Cas6 sequences are highly diverse, and are sometimes found as single genes detached from other Cas or Cmr gene cassettes, we searched for additional, possibly host-encoded, *cas6* genes that might have been previously undetected by blastP. The only protein with a remote similarity is Ssl5096, encoded on plasmid pSYSM. However, Ssl5096 is only 69 amino acids and the similarity to the Cas6 domain is even further restricted, to only 34 residues, indicating that *ssl5096* is likely a pseudogene. We used very relaxed parameters, down to an E-value cut-off of 10^−3^, but did not detect any additional potential homologs.

To address the possibility that CRISPR3 is not functional, we searched among related cyanobacteria for a strain with a system closely related to CRISPR3. We found such a system in *Synechocystis* sp. PCC6714, with high synteny, Cas proteins of 90–100% sequence identity and identical repeat sequences [45]. In fact, the only noteworthy difference between the CRISPR3 systems of both strains are the spacer sequences. Hence, these systems must have been active in spacer acquisition in both strains at least until very recently. In addition, we observed a complex pattern of transcript accumulation as in *Synechocystis* 6803 studied here, indicative of a well-working maturation apparatus. We conclude that CRISPR3 must have been a functional system.

Consequently, we searched for further factors affecting CRISPR3 crRNA accumulation by *in vivo* analysis. We found a Cmr2 homolog to be involved in crRNA accumulation of CRISPR3. Cmr2 proteins possess a GGDEF domain, a classical RNA Recognition Motif (RRM)-fold [46], [47], and have, together with Csm1 and Csx11 proteins, been denoted CRISPR polymerases because of their similarity to the Palm/Cyclase domain [19], [48]. Based on its presence in the CRISPR-Cas effector complex of *Pyrococcus furiosus* that destroys complementary RNAs [18], [29] and its predicted functional domains, Cmr2 was considered the most likely Cmr complex subunit responsible for target RNA cleavage. However, this possibility has recently been challenged when a structural analysis of the Cmr2 homolog from *Pyrococcus furiosus* indicated that it is not the catalytic subunit of the Cmr complex [49]. Our data strongly support a function of Cmr2 in the maturation, regulation of expression, Cmr complex formation or stabilization of CRISPR3 transcripts instead, possibly as the RNA processing endonuclease. In the latter case, the complete loss of transcript accumulation might appear surprising at a first glance, as an effect more like the one observed for the *cas6-1* and *cas6-2a* mutations might have been expected: The accumulation of precursor transcripts but a lack of mature products. However, this result is fully consistent with a recent mathematical model that suggested a competition between specific pre-crRNA processing and non-specific degradation by a yet unidentified nuclease(s), which constitutes a major control element of CRISPR response. This competition determines the steady-state levels of crRNAs [50]. In the case of the *cmr2* mutant, the lack of pre-crRNA processing had to be so severe, that all precursors are prone to rapid degradation. Whereas in the case of the *cas6-2a* mutation, maturation can proceed to a point where an association between the products of binding proteins becomes possible, which leads to their stabilization. Our results are consistent with the reported requirement for a Cmr2/Cas10 component for the accumulation of crRNAs in *Staphylococcus epidermidis in vivo* [28]. For that system, among several possibilities, an activating function on the Cas6 endonuclease was discussed in addition [28]. For CRISPR3 of *Synechocystis* 6803, an activation of Cas6 can be ruled out, however, we need to point out that an alternative function of Cmr2 (to that of an endoribonuclease) in the regulation of expression, Cmr complex formation or stabilization of CRISPR3 transcripts cannot be ruled out at present.

## Materials and Methods

### Culture Media and Growth Conditions

For standard experiments, liquid cultures of *Synechocystis* PCC6803 were grown at 30°C in BG11 medium [51] under continuous illumination with white light of 50 µmol quanta m^−2^ s^−1^ and a continuous stream of air to the desired growth phase (OD_750_ = 0.6–0.8). For the RNA-seq analysis, cultures were initially grown under standard conditions and then subjected to 10 different conditions: (1) exponential growth until an OD_750_ of 0.75; (2) stationary phase until an OD_750_ of 4.724; (3) heat stress, 42°C for 30 min; (4) cold stress, 15°C for 30 min; (5) high light, 470 µMol q s^−1^ m^−2^ for 30 min; (6) dark, no light for 12 h; (7) Fe stress, addition of DFB chelator for 24 h; (8) N depletion, 150 mL of culture were washed 3 times with 100 mM of nitrogen-free BG11 and cultivated further for 12 h; (9) C_i_ depletion, 150 mL of culture were washed 3 times with 100 mL of carbon-free BG11 and cultivated further for 20 h; (10) P depletion, 300 mL of culture were washed 3 times with 100 mL of phosphorus-free BG11 and cultivated further for 12 h.

### *Synechocystis* 6803 Transformation

For each transformation 10 ml of *Synechocystis* 6803 culture (OD_750_ = 0.5–1.0) were centrifuged (4 000 rpm, 20°C, 10 min) and the pellet resuspended in 200 µl BG11 medium. After addition of 1–3 µg plasmid (vector pJet1.2 with adequate insert which should be integrated into the pSYSA plasmid via homologous recombination) the sample was incubated at room temperature for 1 h and then plated on BG11 agar plates without antibiotics. Slightly shaded plates were incubated for 1–2 days at 30°C. For subsequent selection, kanamycin (10 µg/ml) was added to the plates underneath the agar layer. After 3–4 weeks, single colonies were picked and cultivation on plates continued with increasing concentration of antibiotics until full segregation was achieved.

### *Synechocystis* 6803 Conjugation

The triparental mating was used to conjugate *Synechocystis* 6803 cells with a plasmid capable of autonomous replication within *Synechocystis* (pVZ322 - with kanamycin and gentamycin resistance). Overnight cultures of the helper strain *Escherichia coli* J53/RP4 (ampicillin and kanamycin resistance) and the donor strain *Escherichia coli* TOP10 with the plasmid of interest (pVZ322+ insert) were incubated at 37°C. The *E. coli* overnight cultures were diluted 1∶40 with LB medium lacking antibiotics and incubated for 2.5 h at 37°C by agitation at 180 rpm. Cultures were pelleted (2 500 rpm, 20°C, 8 min) and resuspended in 1 ml LB medium. 1 ml helper culture was combined with 1 ml plasmid bearing culture and centrifuged for 5 min at room temperature, 2 500 rpm. The resuspended pellet (in 100 µl LB medium) was incubated for 1 h at 30°C without agitation. Then 800 µl *Synechocystis* 6803 culture (OD_750_ = 0.9) was added and centrifuged again (2 500 rpm, 20°C, 5 min). The pellet was resuspended in 30 µl BG11 medium. A sterile filter (0.45 µm HAFT Millipore - Mixed Cellulose Ester) was placed on a BG11 agar plate supplemented with 5% LB medium (without antibiotics) and the 30 µl conjugation suspension was pipetted on the filter. After an overnight incubation at 30°C with slightly covered plates the filter was rinsed with 400 µl BG11 medium. 50–100 µl of this suspension was plated on BG11 agar plates (with adequate antibiotics Km 10 µg/ml, Gen 1 µg/ml). Further incubation at 30°C should yield conjugants after 1–2 weeks, which can be further selected for by slowly increasing the concentration of antibiotics.

### Knock-out Experiments

To analyse gene functions, selected *cas* genes were knocked out by replacing the gene with a resistance cassette through homologous recombination. The upstream and downstream flanking regions of the corresponding gene were amplified via PCR (for primer sequences, see **Table S1**) and ligated with the resistance cassette, resulting in following construct: upstream flanking region - resistance cassette - downstream flanking region. Three different resistance cassettes were used, providing resistance against the antibiotics kanamycin (from vector puc4K), streptomycin (from vector pRL692) or chloramphenicol (from vector pACYC184). These constructs were ligated into the multiple cloning site of pJet1.2 and the resulting vectors were subsequently used to transform cells of *Synechocystis* 6803.

### RNA Analysis and Hybridization Conditions

100 ml of *Synechocystis* 6803 cultures were harvested through centrifugation (10 000 rpm, 20°C, 8 min). The pellet was resuspended in 1 ml of PGTX [52] and immediately frozen in liquid nitrogen. Samples were then incubated for 5 min at 95°C and put on ice for 5 min. After addition of 1 ml of chloroform/isoamylalcohol (24∶1) and thorough agitation samples were incubated at room temperature for 10 min. Samples were centrifuged with a swing out rotor at 6 000 rpm, 15°C for 15 min. The upper aqueous phase was transferred into a new vial and the same volume of chloroform/isoamylalcohol (24∶1) was added and mixed. Samples were centrifuged as described above and the aqueous phase removed again and combined with an equal volume of isopropanol. After gently inverting the tube RNA was allowed to precipitate over night at −20°C. RNA was pelleted through centrifugation (13 000 rpm, 4°C, 30 min). The pellet was washed with 1 ml of 70% ethanol (13 000 rpm, 4°C, 5 min), allowed to air dry for approximately 10 min and resuspended in 30 µl H_2_O.

8 µg of total RNA per lane were separated on 10% polyacrylamide-urea gels and electroblotted on Hybond-N+ membranes from Amersham. Membranes were prehybridized for at least 30 min at 45°C with hybridization buffer (50% deionized formamide, 7% SDS, 250 mM NaCl, 120 mM NaPi buffer, pH 7.2) in glass tubes under continuous rotation. For northern hybridization, synthetic oligonucleotide probes (**Table S2**) labeled by [^32^P] ATP were used. For 5′ labeling of oligonucleotides with 30 µCi [^32^P] ATP, T4 polynucleotide kinase (Fermentas) was used: 2.5 µl oligonucleotide (10 pmol/µl) and 11.5 µl H_2_O were denatured for 5 min at 95°C and put on ice. After addition of 2 µl 10× buffer A, 3 µl [^32^P] ATP (10 mCi/ml) and 1 µl PNK (10 U/µl) the probe was incubated for 30 min at 37°C and the reaction stopped at 95°C for 5 min. The probe was put on ice and then added to the prehybridizing membrane. Hybridization was done overnight at 45°C. Subsequent washing of the membrane was performed at 40°C with washing solution I (2×SSC, 1% SDS), II (1×SSC, 0.5% SDS) and III (0.1×SSC, 0.1% SDS) for 10 min each. Signals were detected with a storage phosphor screen (Kodak), read on a BIO-RAD Molecular Imager FW system and analyzed with the Quantity One software (BIO-RAD, Germany).

### RNA-seq Data

The cDNA libraries for both datasets were prepared by vertis Biotechnologie, Germany (http://www.vertisbiotech.com/).

For the RNA-seq analysis in dataset A, equal amounts of total RNA from cultures subjected to 10 different conditions (see ‘Culture media and growth conditions’) were mixed and rRNA was depleted using the using the MICROBExpress kit (Ambion). The RNA sample was fragmented with ultrasound (4 pulses of 30 s at 4°C) and then treated with phosphatase. Afterwards, the RNA fragments were re-phosphorylated using T4 polynucleotide kinase and then 3′ poly(A)-tailed using poly(A) polymerase, which was followed by ligation with an RNA adapter to the 5′-phosphate of the RNA fragments. First-strand cDNA synthesis was performed using an oligo(dT)-adapter primer and M-MLV reverse transcriptase. The resulting cDNA was PCR-amplified by 11 cycles to about 20–30 ng/µl using a high fidelity DNA polymerase and primers designed for TruSeq sequencing according to the instructions of Illumina. The cDNA was purified using the Agencourt AMPure XP kit (Beckman Coulter Genomics), analyzed by capillary electrophoresis and size-fractionated for the fraction <450 bp by elution from agarose gels. The cDNA pool was sequenced on an Illumina HiSeq 2000 machine yielding 33,357,164 reads of length 100 nt.

For the RNA-seq data of dataset B, the preparation and analysis of cDNA libraries on a Roche FLX (454) sequencer was previously described as (−) population [36]. A total of 169,360 sequence reads were obtained, from these 129,346 reads were ≥18 nt in length, and 106,018 reads matched to the sequences of the genome or one of the four megaplasmids of *Synechocystis* 6803, including pSYSA [36].

### RNA-seq Mapping

Mapping dataset A: Using the FASTQC analysis tool, we observed an increasingly poor sequencing quality towards read ends in this dataset, possibly due to the poly(A) tails and subsequent adapter sequences (see **Figure S1**). Therefore, in a pre-processing step, the reads were trimmed with respect to their sequencing quality using the fastq_quality_trimmer program from the FASTX-Toolkit version 0.0.13 with the options ″−t 13 −Q 33″. The –Q option is necessary, because the quality scores are used with an ASCII offset of 33 according to the Sanger format. In this way nucleotides were trimmed if they had a quality below 13, which roughly corresponds to an estimated probability that a base call is incorrect greater than 0.05 (p>0.05) [53]. Subsequent to trimming, the dataset was mapped with Segemehl [54] version 0.1.3 with the options “–polyA –prime3 ‘AGATCGGAAGAGCGTCGTGTAGGGAAAGAGTGTAGATCTCGGTGGTCGCCGTATCATT’”, which are the settings for clipping the poly(A) tail and the 3′ Illumina sequencing adapter. Following this mapping procedure, we could successfully map approximately 98% of the original reads to the genome. In total we had over 30 million individual reads.

Mapping of the smaller Dataset B was described in reference [36].

### RNA-seq Read Filtering for CRISPR Expression Profiles

To gain a more accurate picture of the CRISPR array expression, we filtered the original reads to reduce noise. The bulk of noise arises from short sequence reads that cover only the repeat regions and are therefore incorrectly mapped to all repeat instances, obscuring the coverage profiles. Thus we selected reads that mapped with a read quality of 1, an edit distance of 2, were located on the forward strand, and had a unique match. Due to the duplications in CRISPR1 and 2, we also allowed reads for these loci that mapped to two locations. This filtering delivered a cleaned up picture, but did not considerably change the original coverage profiles. These filtered reads are depicted in **Figure 4**.

### Calculation of crRNA Degradation for CRISPR3

Let *i_s_* be the starting and *e_s_* be the ending position of the current spacer *s* and *i_r_* be the starting and *e_r_* be the ending position of the current read *r*. We then considered all reads starting with *i_r_*>*i_s_*−25 and *e_r_*<*e_s_* +10 to represent processed crRNA sequences, read set *C*. Of these reads *C*, we then selected the possibly degraded reads (set *D*) with *i_r_*>*i_s_*−8 and *e_r_*<*e_s_* –10 (we used *e_r_*<*e_s_* –15 for dataset A, because very many reads seemed stable between *e_s_* –10 and *e_s_* –14). It is difficult to select this 3′ cutoff because it is unknown until which length the crRNA is still functional, i.e. can locate its target. The 5′ cutoff is easier due to the fixed cleavage site at *e_s_* –13 (for CRISPR3). The number of potentially degraded crRNA was then normalized by the total number of reads at that crRNA locus, i.e. *degradation ratio = D/C*.

### Ensemble Energies

The RNA structure ensemble energies of each spacer were calculated by RNAfold [55] version 1.8.4 from the Vienna package with the options “-d2 -noLP”. The energies are given as absolute values in kcal/mol. Note that during crRNA maturation, the spacers are trimmed to different lengths. We did not consider these varying lengths that could have an effect on the ensemble energies, however, the most of the spacer remains intact.

### Genome Viewer

To explore the RNA-seq results and display structural properties we used the Integrative Genomics Viewer (IGV) version 2.0.3 [56].

### Sequence of the pSYSA Plasmid

All sequence analyses were done using the publicly available sequence in RefSeq (NC_005230.1) or Genbank (AP004311.1).

### CRISPR-specific Context-based Structure Prediction of Repeats

We followed the procedure described below to produce more accurate structure predictions of repeats that also includes the context sequence of the array.

1. The most probable repeat structure candidates were determined using RNAfold [55], Vienna package version 1.8.4, with parameters “-p –d2 -noLP”, which results in the dot-plot (base-pair probability matrix) in Figure 1B. Omitting the option “-p” calculated the minimum free energy (MFE) structure.
2. To determine the influence of the context sequence on each repeat sequence location, we predicted the structure of the entire CRISPR array. Due to long sequence lengths of CRISPR arrays and unknown contexts due to the intermediate processing steps, we used the local folding approach RNAplfold [57], Vienna package version 1.8.4, options “-noLP –W 150–L 100”. The locality parameter settings for the window size (W) and the maximum base-pair span (L) were taken from reference [58].
3. Subsequently, the sub-matrices for each repeat instance were averaged to form an average dotplot for the repeat structure (see Figure 1C). The dotplot visualizes the average base-pair probabilities for the repeat sequence for all occurrences in the array and includes the influence of the context.
4. The candidate from (1) with the highest structure accuracy in the average dotplot from step (3) (see [58] for a definition of structure accuracy) represents the most probable structure for that CRISPR array. This is the structure that has the highest probability on average across each repeat position. Thus, it is likely to form more frequently at repeat locations than the other candidates. The chosen candidate with the highest accuracy can usually be easily identified in the average dotplot, due to its greater base-pair probabilities and therefore larger dot sizes (blue structure in **Figure 8A**).

Dotplots are read as matrices. Each cell in the top triangle represents a base-pair probability for base i and base j in the bordering sequence. The dimension of each dot is given by the square root of its respective base-pair probability. The bottom triangle represents the base-pairs of the minimum free energy structure, where the dimensions are equal to 1. The average dotplot differs only in the fact that the dots in the upper triangle represent average base-pair probabilities for all sequence occurrences.

## Supporting Information

Figure S1 **Base-pair quality image from the FASTQC program.** (A) We see an increasingly poor sequencing quality towards read ends for the original dataset, possibly due to the poly(A) tails and subsequent adapter sequences. (B) After quality trimming, we see that the read ends with a poor sequencing quality have been removed.
(EPS)

Table S1 **Synthetic oligonucleotides used for knock-out and complementation constructs.** Oligonucleotides named xxx_I_rev contain a single *Age*I site; oligonucleotides named xxx_II_fw contain a single *Fse*I site.
(DOCX)

Table S2 **Synthetic oligonucleotide probes used for northern hybridization (C = CRISPR, S = spacer).**
(DOCX)
