# Supplementary material for: CRISPR-Cas Systems in the Cyanobacterium Synechocystis sp. PCC6803 Exhibit Distinct Processing Pathways Involving at Least Two Cas6 and a Cmr2 Protein
Source: PLoS One. 2013 Feb 18;8(2):e56470. doi: 10.1371/journal.pone.0056470 (PMC3575380; doi:10.1371/journal.pone.0056470)
Supplement: Table S1 — Synthetic oligonucleotides used for knock-out and complementation constructs. Oligonucleotides named xxx_I_rev contain a single AgeI site; oligonucleotides named xxx_II_fw contain a single FseI site. (DOCX) [file pone.0056470.s002.docx]

**Table S1.** Oligonucleotides used for knockout and complementation constructs.

Oligos named xxx_I_rev contain a single *Age*I site, oligonucleotides named xxx_II_fw contain a single *Fse*I site.

| **oligonucleotide** | **sequence (5´→3`)** |
| --- | --- |
| ***Knockout constructs*** | |
| ∆slr7014_I_fw | GAGCGAAGCTGGGGTAATGC |
| ∆slr7014B_I_rev | CAAACTGTGGCCGGCCTCAAACATGATTAATTAG |
| ∆slr7014_II_fw | GGGACCGGTTTCAGTTAACAATCATG |
| ∆slr7014_II_rev | GGATGCTTGAACTCACTCGATAAC |
| ∆slr7068_I_fw | GAATATGAAAATCAATGGCAGAGCAGGG |
| ∆slr7068_I_rev | CCACCGGTTATCCCCACCCCTG |
| ∆slr7068_II_fw | AGCTAGGCCGGCCTTAGCCAATG |
| ∆slr7068_II_rev | GGATCTAAACCTGCCTGGACG |
| ∆sll7090_I_fw | CCGTATCGGTTTTGCCGGAG |
| ∆sll7090_I_rev | GAACCGGTACCGTTACATTAAATTTCCC |
| ∆sll7090_II_fw | GAGGCCGGCCGATTTCCTTTTGGTCTC |
| ∆sll7090_II_rev | GAATATCGTAGGCAATAACATAGAGAAAC |
| ***Complementation constructs*** | |
| petJPro_claI_fw | GAATCGATCTGGCAACTGATTAATC |
| petJPro_xbaI_fw | GCTCTAGAAACTGATTAATCCAC |
| FLAGTTAclaI_rev | ATCGATTTACTTGTCATCGTCAT |
| petJPro_claI_fw2 | GAATCGATatggcaactgattaa |
| 7014-fus_fw | CTTTATCCTTGAAAGGAGAACTAGATACTAATTAATCATGTTTGATG |
| 7014-FLAGStopClaI_rev | ATCGATTCACTTGTCATCGTCATCTTTGTAATCTCCATGATTGTTAACTGAAACCTG |
| 7014-petJProFUS_rev | GATCATCAAACATGATTATTAGTATCTAGTTCTCCTTTCAAGGATAAAG |
| 7068-fus_fw | CTTTATCCTTGAAAGGAGAACTAGGTGGGGATACGTGGTG |
| 7068-FLAGStopXbal_rev | tctagattacttgtcatcgtcatctttgtaatcttgaacattggctaaggccc |
| 7068-petJProFUS_rev | CACCACGTATCCCCACCTAGTTCTCCTTTCAAGGATAAAG |
| 7090-fus_fw | CTTTATCCTTGAAAGGAGAACTACAAAAGGAAATCCGCCTGTG |
| 7090-FLAGStopClal_rev | ATCGATTTACTTGTCATCGTCATCTTTGTAATCATTGGGAAATTTAATGTAACGGTTC |
| 7090-petJProFUS_rev | CACAGGCGGATTTCCTTTTGTAGTTCTCCTTTCAAGGATAAAG |
