# Supplementary material for: CRISPR-Cas Systems in the Cyanobacterium Synechocystis sp. PCC6803 Exhibit Distinct Processing Pathways Involving at Least Two Cas6 and a Cmr2 Protein
Source: PLoS One. 2013 Feb 18;8(2):e56470. doi: 10.1371/journal.pone.0056470 (PMC3575380; doi:10.1371/journal.pone.0056470)
Supplement: Table S2 — Synthetic oligonucleotide probes used for northern hybridization (C = CRISPR, S = spacer). (DOCX) [file pone.0056470.s003.docx]

**Table S2.** Synthetic oligonucleotide probes used for northern hybridization (C = CRISPR, S = spacer).

| oligonucleotide | length (nt) | sequence (5´→3`) |
| --- | --- | --- |
| **CRISPR1** | | |
| C1S1 | 26 | gcattgaaagcgaccgccaggggcac |
| **CRISPR2** | | |
| C2S6 | 29 | ttgtcgagcttagtagtgtggtttctttg |
| **CRISPR3** | | |
| C3S1 | 27 | cattcctaatctaaaggtcaacgccca |
| C3S2 | 27 | cttcaggaactctgtcagctgtggcgc |
